# A comparative study of the effects of Aducanumab and scanning ultrasound on amyloid plaques and behavior in the APP23 mouse model of Alzheimer disease

**DOI:** 10.1186/s13195-021-00809-4

**Published:** 2021-04-09

**Authors:** Gerhard Leinenga, Wee Kiat Koh, Jürgen Götz

**Affiliations:** Clem Jones Centre for Ageing Dementia Research, Queensland Brain Institute, The University of Queensland, Brisbane, QLD 4072 Australia

**Keywords:** Alzheimer’s disease, Blood–brain barrier, Dementia, Amyloid-β, Immunotherapy, Focused ultrasound

## Abstract

**Background:**

Aducanumab is an anti-amyloid-β (Aβ) antibody that achieved reduced amyloid pathology in Alzheimer’s disease (AD) trials; however, it is controversial whether it also improved cognition, which has been suggested would require a sufficiently high cumulative dose of the antibody in the brain. Therapeutic ultrasound, in contrast, has only begun to be investigated in human AD clinical trials. We have previously shown that scanning ultrasound in combination with intravenously injected microbubbles (SUS), which temporarily and safely opens the blood-brain barrier (BBB), removes amyloid and restores cognition in APP23 mice. However, there has been no direct testing of how the effects of SUS compare to immunotherapy or whether a combination therapy is more effective.

**Methods:**

In a study comprising four treatment arms, we tested the efficacy of an Aducanumab analog, Adu, both in comparison to SUS, and as a combination therapy, in APP23 mice (aged 13–22 months), using sham as a control. The active place avoidance (APA) test was used to test spatial memory, and histology and ELISA were used to measure amyloid. Brain antibody levels were also determined.

**Results:**

We found that both Adu and SUS reduced the total plaque area in the hippocampus with no additive effect observed with the combination treatment (SUS + Adu). Whereas in the cortex where there was a trend towards reducing the total plaque area from either Adu or SUS, only the combination treatment yielded a statistically significant decrease in total plaque area compared to sham. Only the SUS and SUS + Adu groups included animals that had their plaque load reduced to below 1% from above 10%. There was a robust improvement in spatial memory for the SUS + Adu group only, and in this group the level of Adu, when measured 3 days post-treatment, was 5-fold higher compared to those mice that received Adu on its own.

Together, these findings suggest that SUS should be considered as a treatment option for AD. Alternatively, a combination trial using Aducanumab together with ultrasound to increase brain levels of the antibody may be warranted.

**Supplementary Information:**

The online version contains supplementary material available at 10.1186/s13195-021-00809-4.

## Background

The deposition of amyloid-β (Aβ) in the brain is considered to be a key initiating step in the development of Alzheimer’s disease (AD). Approaches that either prevent or remove the accumulation of Aβ in the brain have been a focus of research into developing a therapy for this disorder [1–3], with several active and passive immunization strategies being explored in clinical trials to enhance Aβ clearance from the brain.

Aducanumab is an anti-Aβ antibody that targets Aβ aggregates including insoluble fibrils and soluble oligomers, by binding to the amino-terminus of Aβ at residues 3–7 in a shallow pocket in the antibody [4]. This human IgG1 antibody was isolated from the B cells of cognitively healthy elderly humans and has low affinity for monomeric Aβ [5]. In a Biogen-sponsored phase Ib clinical trial (PRIME) of Aducanumab in prodromal and mild AD patients, a striking reduction in amyloid plaques as measured by positron emission tomography (PET) was reported following one year of monthly intravenous antibody infusions at doses ranging from 3 to 10 mg/kg. One of the two phase III trials of Aducanumab, EMERGE, unlike ENGAGE, showed reductions in cognitive decline, possibly reflecting the effects of higher accumulated doses of the antibody [6]. Biogen is currently seeking U.S. Food and Drug Administration (FDA) approval for Aducanumab and may be granted conditional approval of the therapy pending a post-market commitment of a phase IIIB re-dosing trial that has recently been launched. If approved, it will be the first anti-amyloid agent and first antibody treatment for AD. However, it remains to be determined whether Aducanumab is a disease-modifying therapy that achieves significant clinical benefits in AD patients [7]. The interpretation of the cognitive data from these trials is complex, given that dosing was altered or stopped during the trial, and the magnitude of the cognitive effect was relatively small. Sevigny et al [5] demonstrated 50% plaque reduction in 9.5–15.5-month-old amyloid precursor protein (APP) mutant Tg2576 mice after treating with a mouse IgG2a Aducanumab analog; however, the effects of the immunotherapy on behavioral read-outs in mouse models have not been reported.

Therapeutic ultrasound is an alternative strategy for clearing amyloid by transiently opening the blood-brain barrier (BBB) and allowing for the uptake of blood-borne factors and therapeutic agents [8]. Given that ultrasound parameters are highly tunable, this technique can be safely applied to a range of species, including mice [9, 10], dogs [11], sheep [12, 13], and macaques [14, 15] as well as humans [16]. Even without using a therapeutic agent (such as an antibody), repeated opening of the BBB with the scanning ultrasound (SUS) approach in 12 and 22 month-old APP23 mice has been shown to activate microglia, thereby reducing amyloid and improving memory performance [17, 18]. This was shown to be dependent on BBB opening rather than simply applying ultrasound without microbubbles to induce a neuromodulatory effect [19]. The underlying mechanisms of ultrasound-mediated BBB opening have not been fully dissected but involve both facilitated para- and transcellular transport [20]. In preclinical studies, ultrasound has also been used to deliver model molecules of various sizes [21], as well as antibodies [22–24] to the brain.

Here, we sought to compare the efficacy of treatment with an Aducanumab analog (Adu) alone, SUS alone, and a combination of both SUS and Adu in terms of plaque reduction and performance in a spatial memory task.

## Materials and methods

### Study design

**Fig. 1:**
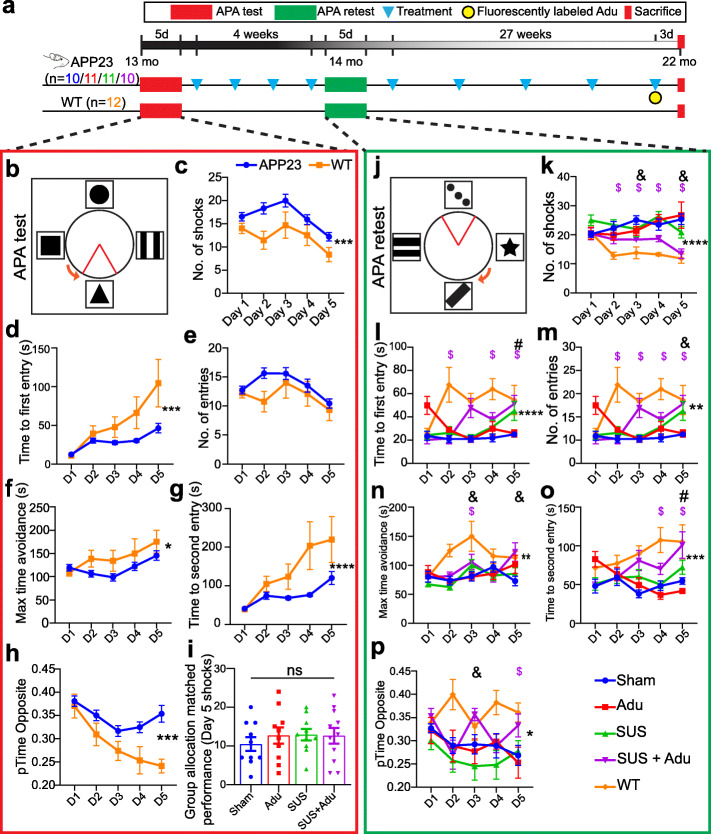
Study overview and results of APA test and retest. Overview of the study with timeline (**a**). In the active place avoidance test (APA) mice must use spatial cues to avoid a shock zone (indicated as a red triangle) (**b**). APP23 mice had impaired performance in the APA test in terms of number of shocks received (**c**), and time to first entry to the shock zone (**d**) as determined by a two-way ANOVA. Although the APP23 mice did not show significant impairment in the measure number of entries (**e**) or maximum time avoidance (of the shock zone) (**f**), they were impaired on the measures time to second entry (**g**) and proportion of time spent in the opposite quadrant to the shock zone (**h**). The mice were then assigned to treatment groups based on matching performance on day 5 of the APA test (**i**). The APA retest was performed after four once-per-week treatments with changes to room cues, shock zone location, and the direction of rotation (**j**). In the post-treatment APA retest an effect of SUS + Adu treatment on number of shocks compared to sham-treated mice was revealed (**k**), whereas treatment with SUS improved time to first entry (**l**). SUS + Adu improved the performance of the mice on the measures number of entries (**m**), and maximum time avoidance (**n**). Time to second entry was improved by SUS (**o**), while SUS + Adu improved the proportion of time spent in the opposite quadrant to the shock zone (**p**). Data are represented as mean ± SEM. Statistical differences: *p < 0.05, **p < 0.01, ***p < 0.001, ****p < 0.0001, $ = simple effect comparing wild-type vs sham *p* < 0.05, # = simple effect comparing SUS vs sham *p* < 0.05, & = simple effect comparing SUS + Adu vs sham *p* < 0.05. Sham *N* = 10, Adu *N* = 11, SUS *N* = 11, SUS + Adu *N* = 10, WT *N* = 12. Data were analyzed with a two-way ANOVA and follow-up Holm-Sidak tests for simple effects

APP23 mice express human APP751 with the Swedish double mutation (KM670/671NL) under the control of the neuron-specific mThy1.2 promoter. As they age, these mice exhibit memory deficits [25], amyloid plaque formation which is initiated in the cortex, and cerebral amyloid angiopathy (CAA) [26]. In this study, APP23 mice, aged 13 months, were assigned to four treatment groups: sham (*N* = 10), SUS (*N* = 11), Adu (5 mg/kg delivered retroorbitally, *N* = 11), or SUS + Adu (5 mg/kg retroorbitally, *N* = 10). Assignment to treatment groups was based on matching performance of spatial memory (number of shocks) on day 5 of the active place avoidance (APA) test. We have previously shown that this approach reduces variability because mice yield similar results when repeatedly tested (as revealed by a main effect of subject) [17], and repeated APA testing of the same mouse can detect the effect of hippocampal injury and exercise, demonstrating the intra-animal validity of this approach [27]. A group of wild-type mice (*N* = 12) was also included. APP23 mice were ranked from those receiving the fewest shocks to those receiving the most shocks on day 5 and were assigned to the four treatment groups (sham, SUS, Adu, SUS + Adu) in rank order. Each group received a total of nine treatments (an APA retest was performed after the fourth treatment), with the final treatment in the Adu and SUS + Adu groups using fluorescently labeled antibody (2.5 mg/kg Alexa Fluor 647-labeled Adu and 2.5 mg/kg unlabeled Adu) (Fig. 1a). Three days after the final treatment, the mice were administered an overdose of sodium pentobarbitone and perfused with phosphate-buffered saline (PBS). The right hemisphere of the brain was fixed in 4% paraformaldehyde for histology, while the cortex and hippocampus of the left hemisphere were dissected and frozen in liquid nitrogen for subsequent analysis. Due to the increased mortality of this strain [28], the numbers of mice surviving to 22 months for histological and biochemical analysis were *N* = 10 sham, *N* = 9 Adu, *N* = 8 SUS, and *N* = 9 SUS + Adu. Assessment of outcomes was performed with the researcher blinded to the treatment group. All animal experimentation was approved by the Animal Ethics Committee of the University of Queensland (approval number QBI/554/17). Sample sizes for the experiment were selected based on our earlier studies [17]. Due to availability, mostly male mice were used (males/females: sham = 9/1, Adu = 8/1, SUS 7/1, SUS + Adu 7/2, Wild-type = 9/3 in the mice that survived to 22 months). We were unable to perform a third APA test as 22-month old APP23 mice are unable to physically perform the task. Data was collected for all mice that survived until the end of the experiment and all data was included.

### SUS equipment

An integrated focused ultrasound system (Therapy Imaging Probe System, TIPS, Philips Research) was used. This system consisted of an annular array transducer with a natural focus of 80 mm, a radius of curvature of 80 mm, a spherical shell of 80 mm with a central opening of 31 mm diameter, a 3D positioning system, and a programmable motorized system to move the ultrasound focus in the x and y planes to cover the entire brain area [17]. A coupler mounted to the transducer was filled with degassed water and placed on the head of the mouse with ultrasound gel for coupling, to ensure unobstructed propagation of the ultrasound to the brain.

### Production of microbubbles

Microbubbles comprising a phospholipid shell and octafluoropropane gas core were prepared in-house. 1,2-distearoyl-*sn*-glycero-3-phosphocholine (DSPC) and 1,2-distearoyl-*sn*-glycero-3-phosphoethanolamine-N-[amino (polyethylene glycol)-2000] (DSPE-PEG2000) (Avanti Polar Lipids) were mixed in a 9:1 M ratio and dissolved in chloroform (Sigma), after which the chloroform solvent was evaporated under vacuum. The dried phospholipid cake was then dissolved in PBS with 10% glycerol to a concentration of 1 mg lipid/ml and heated to 55 °C in a sonicating water bath. The solution was placed in a 1.5 ml glass HPLC vial with the air in the vial replaced with octafluoropropane gas (Arcadophta). The microbubbles were activated on the day of the experiment by agitation of the vial in a dental amalgamator at 4000 rpm for 45 s. Activated microbubbles were measured with a Multisizer 4e coulter counter which reported a mean diameter of 1.885 μm and a concentration of 9.12 × 10^8^ microbubbles/ml. These microbubbles were also observed to be polydisperse under a microscope (Supplementary Figure 1).

### SUS application

Mice were anesthetized with ketamine (90 mg/kg) and xylazine (6 mg/kg), and the hair on their head was shaved and depilated. They were then injected retro-orbitally with 1 μl/g body weight of microbubble solution and placed under the ultrasound transducer with the head immobilized. A heating pad was used to maintain normal body temperature. Parameters for the ultrasound delivery were 1 MHz center frequency, 0.7 MPa peak rarefactional pressure, 10 Hz pulse repetition frequency, 10% duty cycle, and a 6-s sonication time per spot. The focus of the transducer was 1.5 mm × 12 mm in the transverse and axial planes, respectively. The motorized positioning system moved the focus of the transducer array in a grid with 1.5 mm spacing between individual sites of sonication so that ultrasound was delivered sequentially to the entire brain as described previously [17, 18]. Mice typically received a total of 24 spots of sonication in a 6 × 4 raster grid pattern. For the sham treatment, mice received all injections and were placed under the ultrasound transducer, but no ultrasound was emitted. When the animals were treated with Adu antibody, the solution was mixed briefly with the microbubble solution and injected into the retro-orbital sinus before the mouse was placed under the ultrasound transducer. The time between injecting microbubbles and commencing ultrasound delivery was 60 ± 10 s and the duration of sonication was approximately 3 min (total time from microbubble injection approximately 4 min). We assumed that 100% of the antibody reached the circulation where it circulated with a half-life of 2.5 days [5] and that there was no interference from mixing with the microbubbles which have a half-life of 2 min.

### Production of the Aducanumab analog

VH and VL sequences were identified in Biogen Idec’s patent submission for BIIB-037 WO2014089500 A1 and were cloned into mouse IgG2a and kappa pcDNA3.1 vectors (GenScript). Murine chimeric Aducanumab (Adu) was produced using the Expi293 expression system, purified using protein A chromatography and verified to be endotoxin-free by LAL assay (Thermo Fisher).

### Antibody affinity ELISA

The EC_50_ of Adu was determined by direct-binding ELISA. Aβ_1-42_ fibrils were generated by incubating 0.1 mM Aβ_1-42_ peptide (JPT Peptide Technologies) in 10 mM HCl for 3d at 37 °C. A MaxiSorp ELISA plate was coated with 2 μ/ml Aβ_1-42_ fibrils in 0.1 M sodium bicarbonate buffer and then blocked with 1% bovine serum albumin. The EC_50_ was determined by incubating the wells with serial dilutions of Adu, followed by washing and detection of bound Adu with a rabbit anti-mouse horseradish-peroxidase-conjugated antibody (Dako) and 3,3′,5,5′-tetramethylbenzidine substrate. The 6E10 antibody [29] was used as a positive control for Aβ binding and its EC_50_ was determined for comparison with Adu using the same methods (Supplementary Figure 2).

### Antibody labeling

Adu was covalently conjugated with Alexa Fluor 647 dye (Thermo Fisher Scientific) in PBS with 0.1 M sodium bicarbonate as previously described [22]. The protein concentration and degree of labeling were determined by measuring absorbance at 280 nm and 650 nm, respectively.

### Tissue processing

Mice were deeply anesthetized with pentobarbitone before being perfused with 30 ml of PBS, after which their brains were dissected. One hemisphere of the brain was fixed overnight in a solution of 4% wt/vol paraformaldehyde, and then cryoprotected in 30% sucrose and sectioned coronally at 40 μm thickness on a freezing-sliding microtome (SM2000R, Leica). A one-in-eight series of sections was stored in PBS containing 0.01% sodium azide at 4 °C for subsequent staining.

### Assessment of amyloid plaques

For the assessment of amyloid plaque load, an entire one-in-eight series of coronal brain sections taken from the start of the anterior commissure to the ventral hippocampus of one hemisphere at 40 μm thickness was stained using the Campbell-Switzer silver stain protocol that discriminates fibrillar from less aggregated amyloid as previously described [17]. Stained sections were mounted onto microscope slides and imaged with a × 10 objective on a Metafer bright-field VSlide scanner (MetaSystems) using Zeiss Axio Imager Z2. Analysis of amyloid plaque load was performed on all stained sections using ImageJ. Separate regions of interest were drawn around the cortex and dorsal hippocampus. As both black and amber plaques are present in the sections representing different types of amyloid compactness, they were analyzed separately using a color deconvolution method and automated thresholding to distinguish the two types of amyloid plaques. For the analysis of black plaques, a color deconvolution vector was used followed by the MaxEntropy auto thresholding function in ImageJ. As black plaques consist mainly of diffuse fibrils, no size filter was applied. To measure amber plaques, a second color deconvolution vector was used, followed by invert function and automated thresholding using the triangle method in ImageJ, fill-holes function, and a 60-μm^2^ size filter was applied. Using this method, plaque number, total plaque area, average plaque size, and % area covered by plaque were obtained for both the black and amber plaques and summed to give total plaque area for the cortex and hippocampus. We were unable to analyze the hippocampus of one mouse in the Adu-treated group because of folds in the tissue.

### Assessment of cerebral amyloid angiopathy

To assess CAA, a one-in-eight series of Campbell-Switzer silver-stained sections was examined. Regions of interest were drawn manually around areas of CAA in the cortex, which were distinguished from plaques by having a rod-like structure indicative of blood vessels and a diameter greater than 15 μm. Meningeal CAA which has a ring-shaped structure and occurred close to the edge of the section was also measured. The number of CAA deposits per section, the average size, and the % area of the brain sections positive for CAA staining were determined.

### Assessment of cerebral microbleeds

Prussian blue staining was performed using freshly prepared 5% potassium ferrocyanide and 5% hydrochloric acid (Sigma) for 30 min. Cerebral microbleeds were identified at a × 20 magnification as focal clusters of blue hemosiderin deposits which were smaller than 50 μm wide and appeared to have a perivascular location. A randomly selected subset of 5 mice per treatment group were stained and 4 sections were analyzed from each mouse.

### Immunofluorescence labeling

Coronal 40 μm sections were co-stained with the 4G8 antibody against Aβ (1:1000, Covance) and against Iba1 (1:1000 Wako), followed by goat anti-mouse and goat anti-rabbit Alexa Fluor-conjugated secondary antibodies (1:2000, Thermo Fisher). Alexa Fluor 647-conjugated Adu was detected in situ without additional amplification. Sections were cover-slipped and imaged with a fluorescence slide scanner (Metafer).

### Enzyme-linked immunosorbent assay for Aβ

Frozen cortices were homogenized in 10 volumes of a solution containing 50 mM NaCl, 0.2% diethylamine (DEA) with complete protease inhibitors, and Dounce homogenized by passing through 19 and 27 gauge needles. The samples were then centrifuged at 21,000×*g* for 90 min at 4 °C. The supernatant was retained as the DEA-extracted soluble Aβ fraction. The remaining pellets were resuspended in 10 volumes of 5 M guanidine HCl, sonicated, and centrifuged at 21,000×*g* for 30 min at 4 °C. The resultant supernatant was retained as the guanidine-extracted insoluble Aβ fraction. The concentrations of Aβ_40_ and Aβ_42_ were determined in brain lysates using ELISA kits according to the manufacturer’s instructions (human Aβ_40_ and Aβ_42_ brain ELISA, Merck).

### Active place avoidance test

The active place avoidance (APA) task is a test of hippocampus-dependent spatial learning. We used a repeated APA paradigm, where mice were tested in the APA one time and the performance of each mouse was used to assign that mouse to one of four treatment groups. This was done by ranking all the mice based on their performance and assigning them to the four groups in order so that the APA performance of each treatment group was the same. Following this, mice received either sham, SUS, Adu, or SUS + Adu treatment and 3 days after the last treatment mice were retested in the APA to assess whether there was an improvement in APA performance due to the treatment the mouse had received. For each APA test, APP23 mice and non-transgenic littermate controls were tested over 6 days in a rotating elevated arena (Bio-Signal group) that had a grid floor and a 32-cm-high clear plastic circular fence enclosing a total diameter of 77 cm. High-contrast visual cues were present on the walls of the testing room. The arena and floor were rotated at a speed of 0.75 rpm, with a mild shock (500 ms, 60 Hz, 0.5 mA) being delivered through the grid floor each time the animal entered a 60-degree shock zone, and then every 1500 ms until the animal left the shock zone. The shock zone was maintained at a constant position in relation to the room. Recorded tracks were analyzed with Track Analysis software (Bio-Signal group). A habituation session was performed 24 h before the first training session during which the animals were allowed to explore the rotating arena for 5 min without receiving any shocks. A total of five training sessions were held on consecutive days, one per day with a duration of 10 min. After day 5 of the first APA (test), APP23 mice were divided into four groups with mice matched so that the performance (number of shocks) of the four groups of mice on day 5 of the task was the same, for the retest. Following four once-a-week SUS or Adu treatments, the mice underwent the APA test again (reversal learning). The retest was held in the same room as the initial test. However, the shock zone was switched to the opposite side of the arena, the visual cues were replaced with different ones, and the platform was rotated clockwise rather than counterclockwise. The number of shocks, numbers of entries to the shock zone, time to first entry, time to second entry, and proportion of time spent in the opposite quadrant of the shock zone for sham, SUS, Adu, and SUS + Adu-treated groups were compared over the days of testing.

### Statistical analysis

Statistical analyses were conducted with Prism 8 software (GraphPad). Values were always reported as mean ± SEM. One-way ANOVA followed by the Holm-Sidak multiple comparisons test, or *t* test was used for all comparisons except APA analyses where two-way ANOVA with day as a repeated measures factor and group as a between subjects factor was performed, followed by the Holm-Sidak multiple comparisons test for simple effects to compare group performance on different days. The model assumption of equal variances was tested by Brown-Forsyth or Bartlett tests, and the assumption of normality was tested by Kolmogorov-Smirnov tests and by inspecting residuals with QQ plots. All observations were independent, with allocation to groups based on active place avoidance where mice were ranked on performance and assigned to one of the four groups (sham, SUS, Adu, SUS + Adu) in order of number of shocks on day 5 listed from most to least shocks.

## Results

### Generation of Aducanumab analog and application

The Aducanumab analog Adu was generated by grafting the VH and VL chains of Aducanumab onto a mouse IgG backbone and expressing this in Expi293 cells. We then established that the affinity of Adu to fibrillar Aβ_42_ (EC_50_ 81.7 pM) was similar to that published earlier for Aducanumab (EC_50_ 100 pM) [5]. In comparison, the 6E10 antibody had an EC50 of 1.18 nM for Aβ_42_ fibrils (Supplementary Figure 2). Next, 13-month-old APP23 mice were divided into four groups (sham/Adu/SUS/SUS + Adu) based on matching performance on day 5 (final day) of the initial APA test. A dose of 5 mg/kg Adu was given for each treatment, except for the last treatment where a mixture of 2.5 mg/kg unlabeled Adu and 2.5 mg/kg Alexa Fluor 647-labeled Adu was administered. The mice were initially treated once a week for 4 weeks, after which they were re-tested in the APA. From 15 to 22 months of age, the mice were subsequently treated five times, and then sacrificed three days following the last treatment, resulting in a total of nine treatments (Fig. 1a).

### Aducanumab analog, when delivered by SUS, improves spatial memory performance

In the current study, we compared the effect of delivering the murine chimeric IgG2a Aducanumab analog, Adu, with a SUS treatment, using plaque burden and behavior as the major read-outs. We also assessed a combination treatment (SUS + Adu). Additional comparisons were made by including sham-treated mice, as well as untreated wild-type littermate controls.

We first tested 13 month-old APP23 mice and their wild-type littermates in the APA test of hippocampus-dependent spatial learning in which the animals must use visual cues to learn to avoid a shock zone located in a rotating arena (Fig. 1b). Spatial learning was not assessed in the alternative Morris water maze test because this test is stressful to mice, and aged mice are poor swimmers [30, 31]. To determine the effect of each treatment protocol on spatial memory function, an APA test consisting of 5 training days with a single 10 min training session each day was performed following habituation to the arena in one 5 min session the day before the first training day. A two-way ANOVA based on the number of shocks that were received revealed a significant effect of day of testing, indicating that learning had occurred (*F*_4,208_) = 5.728, *p* = 0.0003. There was also a significant effect of genotype, with APP23 mice receiving more shocks than their wild-type littermates (*F*_1, 52_) = 6.278, *p* = 0.0154 (Fig. 1c). Similarly, based on the measure of time to first entry of the shock zone, there was a significant effect of day, with mice showing longer latencies to the first entrance as the number of training days increased (*F*_4,208_) = 7.586, *p* = 0.0007. Wild-type mice exhibited longer latencies to enter the shock zone over the days of testing and there was a significant effect of genotype on time to first entry (*F*_*1.52*_) *=* 5.950, *p* = 0.0182 (Fig. 1d). Wild-type and APP23 mice did not differ; however, on number of entries (Fig. 1e) or maximum time of avoidance of the shock zone (Fig. 1f). APP23 mice performed significantly worse on the measures “time to second entry” (Fig. 1g) and “proportion of time spent in the quadrant opposite to the shocked quadrant” (Fig. 1h). The APA performance of the APP23 mice varied significantly so they were assigned to each of the four treatment groups based on matching performance in terms of the number of shocks received on day 5 of the APA to reduce any differences in performance between treatment groups to more readily detect any improvement (Fig. 1i).

Before retesting in the APA, mice were treated once a week for 4 weeks. For the retest, the shock zone was shifted by 180°, the cues in the room were changed, and the arena rotated in the opposite direction. To perform well in the retest, the mice needed to update their spatial learning in order to learn the new shock zone location (Fig. 1j). A two-way ANOVA with group as a between-subjects factor and day as a repeated measures factor revealed a significant effect of treatment group on number of shocks received (*F*_4,47_ = 8.5, *p* < 0.0001). Follow-up multiple comparisons tests showed that SUS + Adu-treated mice received significantly fewer shocks than sham-treated control mice on days 3 (*p* = 0.0295) and 5 (*p* = 0.0005) (Fig. 1k). Comparison of the Adu-treated to the SUS + Adu-treated mice by two-way ANOVA revealed that the combination treatment resulted in significantly improved performance over Adu alone in terms of number of shocks received during the test (*F*_1,17_ = 6.23, *p* = 0.0231). A two-way ANOVA revealed a significant main effect of group (*F*_4,47_ = 6.8, *p* = 0.0002) on time to first entry into the shock zone, with a follow-up multiple comparisons test showing that SUS-treated mice had a longer latency to enter the shock zone on day 5 of the APA task (*p* = 0.024) (Fig. 1l). SUS + Adu-treated mice performed significantly better than sham-treated mice on the measures number of entries (Fig. 1m), and maximum time of avoidance (Fig. 1n) SUS-treated mice showed improvement on the measure time to second entry (Fig. 1o). SUS + Adu-treated mice showed improvement in the measure proportion of time spent in the quadrant opposite the shock zone (Fig. 1p). These results demonstrate that APP23 mice exhibit an improvement in spatial memory when treated with a combination of SUS and Adu, and on some measures SUS alone improved performance in the APA.

### Comparison of plaque reduction in the cortex for the different treatment groups

**Fig. 2:**
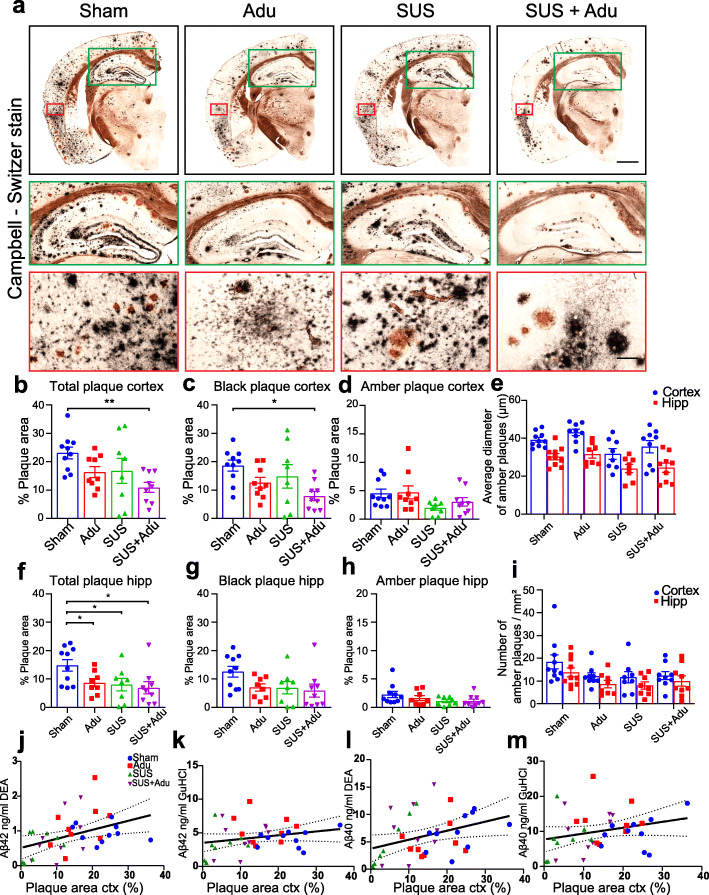
Treatment strategies reduce plaques in APP23 mice. **a** Representative Campbell-Switzer silver staining for amyloid plaques in the four treatment groups. Plaques stained black are more diffuse, whereas amber plaques are compact and discrete. The black box shows the entire hemisphere (scale bars 1 mm). Insert outlined in green shows higher magnification view of dorsal hippocampus (scale bars 500 μm), and the red inset shows higher magnification image of cortex overlying the hippocampus (scale bars, 100 μm). **b** There was a significant reduction of plaque burden in the cortex of SUS + Adu-treated mice, driven largely by reduction in black plaques (**c**) as area, number and size of amber plaque was less affected by treatment (**d**, **e**, **i**). Plaque load in the hippocampus was reduced by Adu, SUS and SUS + Adu (**f**), with hippocampal black plaques (**g**) and amber plaques (**h**) analyzed separately. A significant correlation was found between amyloid plaque burden measured by histology and Aβ levels in cortical lysate measured by ELISA (**j–m**). Data are represented as mean ± SEM. Statistical differences: *p < 0.05, **p < 0.01, ***p < 0.001. Data were analyzed with a one-way ANOVA and follow-up Holm-Sidak tests. Sham *N* = 10, Adu *N* = 9, SUS *N* = 8, SUS + Adu *N* = 9

Following APA testing to ascertain the effects of four once-per-week treatments on spatial memory performance, APP23 mice had five further treatment sessions between the age of 15 and 22 months in order to determine whether SUS, Adu alone, or the combination resulted in robust plaque removal, even at older ages when plaque burden is maximal as the animals were no longer able to physically perform the APA task. The mice were sacrificed at 22 months of age, 3 days after the last treatment, and one hemisphere was processed for histology to identify plaques. We performed Campbell-Switzer staining, which can differentiate diffuse and compact species of amyloid plaques in the brain and is not confounded by the binding of Adu to Aβ. This revealed a reduction in the total plaque area when comparing the treatment groups to sham controls (Fig. 2a). We calculated the percentage area occupied by plaque for two regions of interest, the cortex and the hippocampus, in 15–20 sections per mouse, assessing plaque burden in a one-in-eight series of sections along the rostral-caudal axis starting from the anterior commissure and ending at the ventral hippocampus. Analysis of cortical plaque burden in the different groups revealed an effect of treatment (*F*_3,31_ = 3.78, *p* = 0.02). A follow-up Holm-Sidak test found that combined SUS + Adu treatment resulted in a statistically significant 52% plaque reduction in the cortex of these mice compared to sham (*p* = 0.0066). At 22 months of age, APP23 mice have a severe plaque burden, with diffuse and compact plaques occupying 23% of the cortex in the sham-treated mice, compared to 16% of the cortex in mice administered Adu, 17% in mice administered SUS only, and 11% in mice which received SUS + Adu treatment (Fig. 2b). As an additional analysis, the SUS + Adu treatment was found to be superior to Adu alone in reducing plaque burden (one-tailed *t* test, *p* = 0.029). As the Campbell-Switzer silver stain differentiates diffuse plaques which stained black with a cotton wool appearance, from compact plaques which stain amber (Fig. 2a), we also analyzed these plaques separately using a color deconvolution method in ImageJ. The results of this analysis revealed that the reduction in total plaque area was largely driven by a reduction in the total area of black plaque which occupied 18.60% of the cortical area in sham-treated mice compared to 7.93% in the SUS + Adu-treated animals (*p* = 0.0119) (Fig. 2c). In contrast, we found no significant difference between the treatment groups based on the area, number, or size of amber plaques (Fig. 2d,e,i).

### Aducanumab analog, SUS, and the combination therapy all effectively reduce amyloid plaques in the hippocampus of APP23 mice

In APP23 mice, plaque formation is initiated in the cortex and then proceeds to the hippocampus. When we analyzed the total plaque burden in the hippocampus, we found a significant effect of treatment (F_3,31_ = 3.44, *p* = 0.03, one-way ANOVA). All three treatments (Adu, SUS and SUS + Adu) led to a significant reduction in total plaque area in the hippocampus compared to that in sham-treated APP23 mice (Fig. 2f). The sham-treated mice had a hippocampal plaque burden of 14.84% vs 8.68% for Adu-treated mice (*p* = 0.0432), 8.04% for SUS-treated mice (*p* = 0.043), and 6.92% for SUS + Adu-treated mice (*p* = 0.022). However, unlike the effects seen in the cortex, the reduction in total plaque in the hippocampus was not disproportionately driven by a reduction in black plaque (*F*_3,30_ = 2.43, *p* = 0.08) (Fig. 2g) compared to amber plaque reduction (*F*_3,30_ = 1.80 *p* = 0.17) (Fig. 2e-i), as it was only when total plaque burden was analyzed that statistically significant reductions were found. These results show that the effect of SUS on plaque burden in the hippocampus is comparable to that of Adu and the combination treatment provided no additive effect.

### Effects of treatment on amyloid-β species

We next performed ELISA measurements of Aβ40 and Aβ42 species from the lysate of one cortex, fractionating proteins into a DEA fraction containing soluble proteins and a guanidine fraction containing insoluble proteins. The levels of Aβ42 and Aβ40 in the DEA soluble fraction were significantly correlated to plaque burden as measured by Campbell-Switzer silver staining of the other hemisphere (*R*^2^ = 0.17, *p* = 0.014 and *R*^2^ = 0.13, *p* = 0.030 respectively) (Fig. 2j, l). The levels of Aβ42 and Aβ40 in the guanidine fraction containing insoluble Aβ did not correlate significantly with plaque burden as measured by histology, most likely because most of the material stained by Campbell-Switzer silver staining is soluble in DEA (Fig. 2k, m).

### Aducanumab analog does not affect cerebral amyloid angiopathy in APP23 mice

**Fig. 3:**
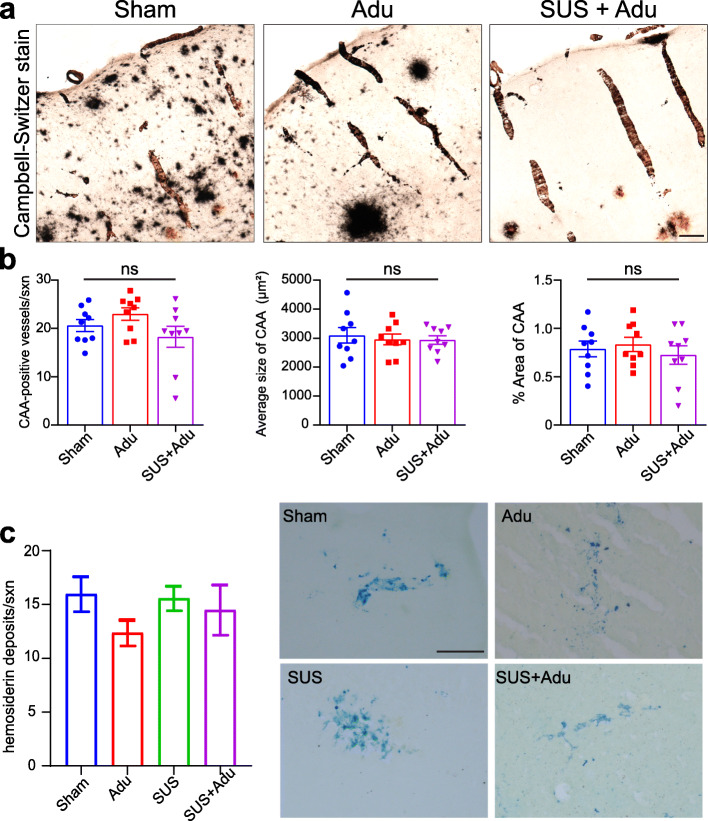
Aducanumab analog does not affect levels of cerebral amyloid angiopathy (CAA) or microhemorrhages in APP23 mice. **a** Representative Campbell-Switzer silver staining shows CAA in the cortex identified by a rod-like appearance, as well as meningeal CAA identified as open circles on top of the cortex. **b** Adu, whether administered with or without SUS, had no effect on the number of CAA-affected vessels, average size, or percent area occupied by CAA. **c** Adu, whether administered by itself or with SUS, had no effect on the number of microhemorrhages detected by Prussian blue staining in 22-month old APP23 mice. Data are represented as mean ± SEM. Statistical differences: *p < 0.05. Data were analyzed with a one-way ANOVA and *t* test. Scale bar 200 μm

We also investigated whether there was any effect of treatment with SUS, Adu, or the combination of the two on CAA. APP23 mice exhibit amyloid deposition on the vasculature with advanced age [32]. We found that there was no effect of Adu or a combination of both on the number of blood vessels that were Campbell-Switzer positive and mice in all groups had significant deposition of amyloid on blood vessels (Fig. 3a). An average of twenty vessels were Campbell-Switzer positive per section, with an average of 0.75% of the total area of the cortex taken up by amyloid-laden blood vessels, and this did not differ between the groups (Fig. 3b).

### Aducanumab does not increase the number of microhemorrhages in APP23 mice

We also investigated whether Adu or SUS + Adu treatment increased the occurrence of microhemorrhages as detected by Perl’s Prussian blue staining for clusters of hemosiderin deposits (considered as a single microhemorrhage) and found that although microbleeds were common in APP23 mice, they did not increase in response to treatment (*F*_3,16_ = 0.94, *p* = 0.44, one-way ANOVA) (Fig. 3b).

### SUS markedly increases the amount of the Aducanumab analog in the brain

**Fig. 4:**
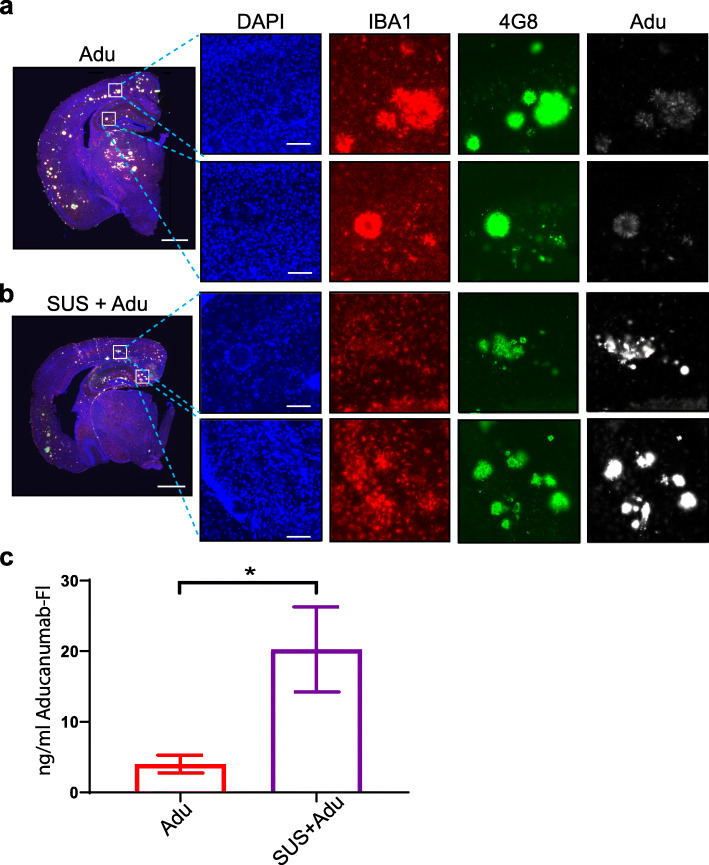
Scanning ultrasound (SUS) increases the levels of the Aducanumab analog in the brain. **a** Fluorescently labeled Adu is detectable in the whole brain (scale bars 1 mm) and when visualized at higher magnification in the cortex and hippocampus (scale bars 100 μm). In APP23 mice treated with Adu alone, the fluorescent Adu is bound to plaques, which were immunolabeled with 4G8 antibody. **b** The levels of Adu were higher when Adu was delivered together with SUS in SUS + Adu-treated mice. The amyloid plaques in SUS + Adu-treated mice were decorated all over with Adu, whereas in mice treated with Adu alone the Adu is mainly confined to the outsides of the plaques. Microglia as identified by IBA1 immunostaining were located near plaques which have Adu bound to them. **c** The levels of fluorescent antibody in the cortical brain lysate was greatly increased in the SUS + Adu group compared to the Adu group. Data are represented as mean ± SEM. Statistical differences: *p < 0.05. Data were analyzed with *t* test. Adu *N* = 9, SUS + Adu *N* = 9

We also sought to determine the extent to which SUS was able to increase the amount of Adu in the brain. To investigate this we labeled Adu with Alexa Fluor 647 and injected the mice with 2.5 mg/kg of the fluorescently labeled Adu and 2.5 mg/kg unlabeled Adu at the last treatment session. In mice treated with Adu alone, fluorescently labeled Adu was faintly detectable by fluorescence microscopy and mainly confined to the outside edge of plaques (Fig. 4a). In contrast, in SUS + Adu mice, fluorescently labeled Adu decorated the entirety of plaques and was easily detectable (Fig. 4b). We also analyzed a subset of 5 mice per group to determine the area of the cortex that was positive for fluorescent Adu, revealing that 0.36% of the cortex in Adu-treated mice was positive compared to 1.59% in SUS + Adu mice (*p* = 0.0096, *t* test). We also detected fluorescently-labeled Adu in mice injected with Adu and SUS + Adu in the cortical lysate and found that levels were 4.32 ng/ml on average in the Adu group compared to 21.77 ng/ml in the SUS + Adu-treated group (*p* = 0.0175, *t* test) (Fig. 4c).

## Discussion

Amyloid-targeted immunization strategies in AD have a long history, whereas therapeutic ultrasound has only recently been explored as a treatment modality. Our earlier studies and those of others have revealed that ultrasound in combination with microbubbles, but in the absence of a therapeutic agent, can clear protein aggregates such as the hallmark lesions of AD, Aβ plaques [17, 18, 33, 34], and tau-containing neurofibrillary tangles [20, 22, 35]. We have also previously shown that the application of SUS achieves BBB opening throughout the brain [17] and, consequently, results in higher brain concentrations of antibodies in an IgG format, with a 19-fold higher concentration of IgG reaching the brain as compared to peripheral injection without SUS [23]. Studies by us in tau transgenic mice [22, 23] and work by others [24, 36] have suggested that ultrasound can also be used as an effective drug delivery tool to increase the level of antibodies in the AD brain.

Here, we used a multi-arm study, in which we compared the effects of SUS, the Aducanumab analog, Adu, delivered peripherally, and Adu delivered to the brain using SUS in APP23 mice with plaque pathology, using a sham treatment and wild-type mice as controls. In our study, SUS treatment had comparable effects to Adu treatment on plaque burden and behavior. We further found that in our treatment paradigm (nine treatments from age 13 to 22 months of age), Adu delivered across the BBB with SUS produced a more marked reduction in the amyloid plaque burden in the cortex of 22 month-old APP23 mice compared to the effects of either the antibody or SUS alone, concomitant with improvements in memory. For the first time, we report data on the effect of an Aducanumab analog in a spatial memory task in plaque-bearing AD model mice. We initially performed the APA test of spatial memory and learning in 13 month-old APP23 mice to obtain a baseline, and then divided the mice into treatment groups based on their performance, which we reasoned would allow us to achieve greater power to detect improvements caused by the treatment due to reduced variability between the groups. We treated mice weekly for 4 weeks and then repeated the APA test in which mice had to learn new spatial cues to avoid the shock zone. This experimental design allowed us to detect an improvement in mice treated with the combination of SUS + Adu compared to sham-treated mice and to detect improved performance in mice treated with the combination compared to mice treated with Adu alone.

We were also interested in the effect of treatment on amyloid plaque burden, specifically at an advanced age (22 months old) in mice when plaque burden is more similar to that of an early AD patient. Interestingly, plaque reduction in the hippocampus could be achieved with any of the three treatments (SUS, Adu and the combination), possibly reflecting the lower degree and later appearance of pathology in this brain area. Somewhat surprisingly, we did not see an improvement in CAA; however, this is in line with findings by Sevigny et al. [5] and could be due to efflux of Aβ_40_ and Aβ_42_ leading to relocation of parenchymal plaques to the vasculature. In clinical trials, microbleeds detectable with MRI occurred in less than 10 % of patients [5]; however, in this study, Aducanumab was not found to increase the occurrence of microbleeds in APP23 mice which could be due to the lower cumulative dose or differences between the parent antibody and the chimeric mouse IgG2a antibody, Adu, used in this study. Spatial memory was improved in 14-month-old APP23 mice following four weekly treatments with a combination of Adu and SUS. We have previously shown that a combination therapy using SUS and an anti-tau antibody in IgG format increased the uptake 19-fold when measured one hour after treatment [23]. In line with these findings, in this study we observed a fivefold increased levels of Adu in this study when administered in combination with SUS, which was measured at a three day time point. The total increased uptake would likely be higher if measured at earlier time points post-injection, as IgG is cleared from the CNS or taken up by microglia as time progresses.

Delivering higher amounts of an anti-Aβ antibody into the brain should increase the efficacy of immunotherapy. This could in principle be achieved by strategies that bypass the BBB such as direct injection into the CNS. In one study, intracranial delivery of Aducanumab by topical application to the cortex was found to rapidly clear plaques in 22-month-old Tg2576 mice, whereas peripheral injections at 10 mg/kg repeated weekly from 18 to 24 months of age proved ineffective at clearing plaques, although they did restore physiological levels of intraneuronal calcium [37]. In contrast, plaque reduction was reported by Sevigny and colleagues when Aducanumab was delivered peripherally in Tg2576 mice. Weekly 10 mg/kg injections begun at 9.5 months of age reduced plaque burden by 50% at 15.5 months, suggesting that Aducanumab treatment may be less effective at removing plaques in mice which already carry a substantial plaque burden compared to preventing plaque formation [5]. Our results show that administering a much lower cumulative dose of an Aducanumab analog than these authors used was ineffective at clearing plaques in the cortex of APP23 mice when treatment was commenced at 13 months of age; however, hippocampal plaques which develop at a more advanced age were reduced. Greater plaque reduction in the hippocampus could also be due to differences in the cerebral vasculature that result in greater drug delivery in the hippocampus than cortex [22]. In contrast to peripheral injections alone, delivery of the Adu using SUS led to a reduction in both cortical and hippocampal plaques, concomitant with increased brain levels of the antibody.

Efforts are currently underway in several laboratories to develop therapeutic ultrasound into a treatment modality for AD and other brain diseases, with ongoing clinical trials using an FDA-approved focused ultrasound system (ExAblate Neuro, Insightec) [16, 38] and implanted transducers [39] (Sonocloud, Carthera). These studies are applying ultrasound with microbubbles, but without a therapeutic agent such as an anti-Aβ antibody, with safe and effective BBB opening being used as primary endpoints, and Aβ clearance as a secondary endpoint. There are clearly several obstacles ahead such as being able to open a large enough brain area repeatedly and safely [8, 40]. Use of therapeutic ultrasound to open the BBB offers the possibility of achieving better brain uptake of a drug that has shown evidence of clinical efficacy, such as Aducanumab [5]. There is also the possibility of reducing the level of antibody that needs to be administered to achieve the same therapeutic outcome, by using ultrasound. This study provides further evidence also for the use of ultrasound opening of the BBB without a therapeutic agent by showing it is not inferior to an anti-Aβ immunotherapy and potentially works through the mechanism of increasing microglial phagocytosis of Aβ [5, 17]. More importantly, we believe that ultrasound can be combined with therapeutic agents (such as an anti-tau antibody and an anti-Aβ antibody) at levels below a safety threshold. What has not been discussed here is the potential of applying ultrasound to target the brain in either a global or a more focused manner. We anticipate that once therapeutic ultrasound has overcome the critical approval hurdles, it may develop into a highly versatile and effective treatment therapy not only for AD but also for other brain diseases.

## Limitations

It must be acknowledged that the current study has some limitations. APP23 mice exhibit significant variability in plaque burden and behavior between animals. The strain is also characterized by a > 40% mortality which presented a challenge in obtaining the high numbers of mice required for the multiple treatment arms. We also used only one APP transgenic mouse strain, and this strain lacks a tau pathology which could limit the clinical relevance of our findings. In addition, most of the mice were male, as a result of which we cannot rule out the possibility that there could be differences in the efficacy of treatment in females and this deserves further study as sex differences in APP23 mice pathology have been reported [41]. Due to the advanced age of the mouse cohort at the end of the treatment period, memory function could not be assessed for a third time. Moreover, only one cognitive test was used to assess spatial memory. Several mice also died prematurely which was due to the premature lethality phenotype of APP23 mice, most likely due to excitotoxicity [28]. We did not observe any differences in deaths between groups, and the age at death did not differ between groups, appearing random. The nature of these deaths and the age at which the treatments were performed precluded detailed investigations of the lethality phenotype. In addition, we only tested one dose of Adu which at 5 mg/kg was lower than the maximum dose tested in the EMERGE and ENGAGE clinical trials of 10 mg/kg.

## Conclusions

An effective therapy for AD would reduce amyloid load and improve cognition. Here, we show that an Aducanumab analog, Adu or SUS alone have a comparable ability to reduce amyloid levels. The combination of SUS and Adu also improves cognitive function as measured by the APA test, suggesting that a trial using Aducanumab in combination with ultrasound to open the BBB has merit as this approach may lead to increased brain levels of Aducanumab. Our data add to the growing literature on applying therapeutic ultrasound either on its own or in combination to treat brain diseases such as AD.

## Supplementary Information

**Additional file 1: Supplementary Figure 1.** Characterization of microbubbles. (a) In-house prepared microbubbles were analyzed by Coulter Counter and number of microbubbles per ml with size displayed. (b) Microbubbles were observed under a microscope at 20x magnification. Scale bar 10 μm.

**Additional file 2: Supplementary Figure 2.** Affinity measurement of Aducanuman analog, Adu. The affinity of Adu for fibrillar Aβ_42_ was determined by ELISA and compared to the antibody 6E10.

## Data Availability

The authors will make data available upon reasonable request.

## Abbreviations

AD: Alzheimer’s disease
SUS: Scanning ultrasound
Aβ: Amyloid beta
ELISA: Enzyme linked immunosorbent assay
Iba1: Ionized calcium binding adaptor molecule 1
IgG: Immunoglobulin
Adu: Aducanumab mouse IgG2a chimeric analogue
Tg: Transgenic
WT: Wild-type
