# Supplementary figures and images for: A comparative study of the effects of Aducanumab and scanning ultrasound on amyloid plaques and behavior in the APP23 mouse model of Alzheimer disease
Source: Alzheimers Res Ther. 2021 Apr 9;13:76. doi: 10.1186/s13195-021-00809-4 (PMC8035770; doi:10.1186/s13195-021-00809-4)

# Supplementary Figure 1

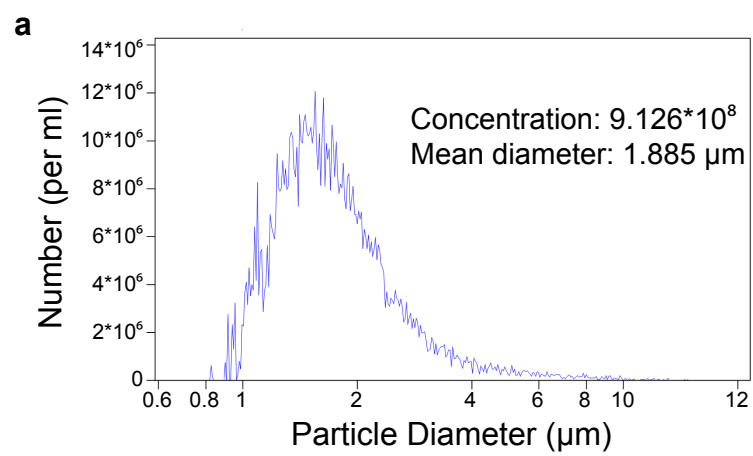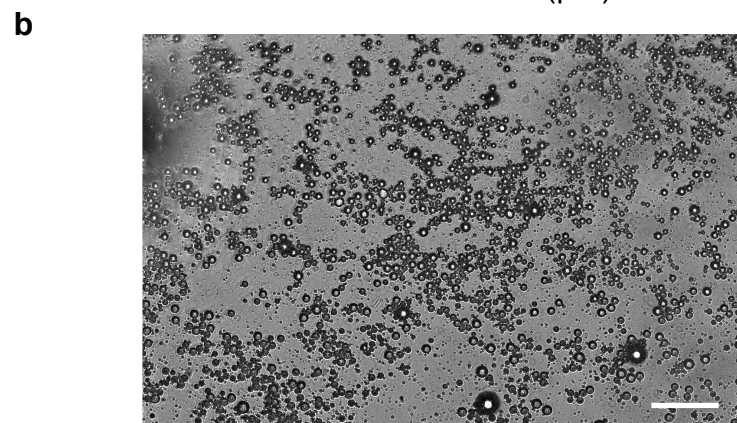

Supplement: Supplementary file 1 — Additional file 1: Supplementary Figure 1. Characterization of microbubbles. (a) In-house prepared microbubbles were analyzed by Coulter Counter and number of microbubbles per ml with size displayed. (b) Microbubbles were observed under a microscope at 20x magnification. Scale bar 10 μm. [file 13195_2021_809_MOESM1_ESM.pdf]

# Supplementary Figure 2

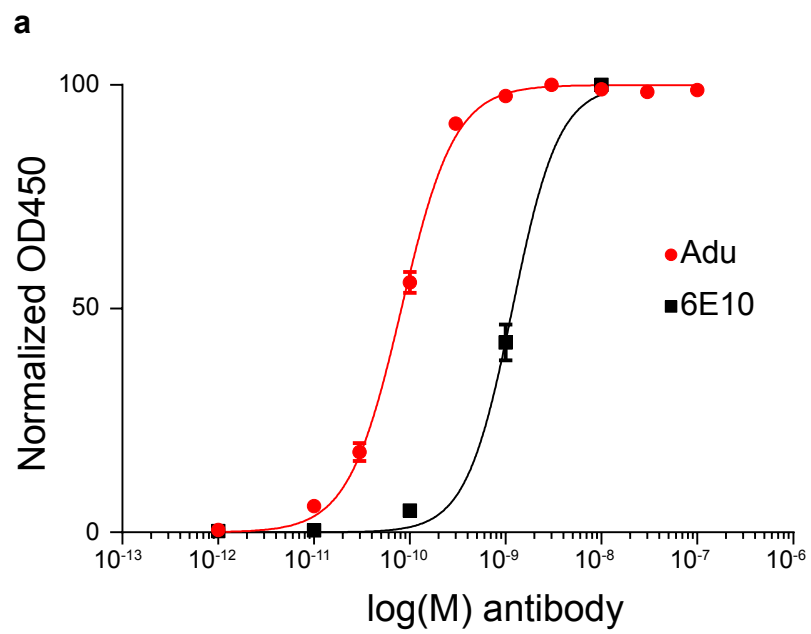

Supplement: Supplementary file 2 — Additional file 2: Supplementary Figure 2. Affinity measurement of Aducanuman analog, Adu. The affinity of Adu for fibrillar Aβ42 was determined by ELISA and compared to the antibody 6E10. [file 13195_2021_809_MOESM2_ESM.pdf]
